# How to Do Empirical Political Philosophy: A Case Study of Miller’s Argument for Needs-Based Justice

**DOI:** 10.1007/s10670-023-00747-7

**Published:** 2023-11-14

**Authors:** Thomas Pölzler

**Affiliations:** Department of Philosophy, University of Graz, Attemsgasse 25/II, 8010 Graz, Austria

## Abstract

In recent years an increasing number of political philosophers have begun to ground their arguments in empirical evidence. I investigate this novel approach by way of example. The object of my case study is David Miller’s renewed empirical argument for a needs-based principle of justice. First, I introduce Miller’s argument. Then I raise four worries about the application of his methodology that give rise to corresponding general recommendations for how to do empirical political philosophy. Proponents of this approach should take care to (1) check for inappropriately narrow (and broad) samples, (2) verify studies’ relevance for their empirical hypotheses, (3) adjust their confidence to the available empirical evidence, and (4) properly integrate their hypotheses into their philosophical theorizing.

## Introduction

Political philosophy has traditionally mainly been conducted from the proverbial philosopher’s armchair.^1^ Only in recent decades a methodological shift or expansion has taken place. Following a general trend in philosophy, an increasing number of political philosophers have begun to ground their arguments, at least *inter alia*, in empirical evidence. Many have in particular appealed to evidence about public opinion, i.e., about the general population’s judgments about normative matters (such as their judgments about justice) (e.g., Brock, 2005, 2009; Bauer et al. 2022; Hassoun, 2009; Lindauer, 2020; Mulligan, 2018; Wolff and de-Shalit 2007).

In other areas of philosophy, the prospects of science-based arguments have been investigated extensively (e.g., May, 2018; Pölzler, 2018; Rose, 2018; Wagenknecht et al., 2015). Researchers have criticized these arguments both on philosophical grounds (Do the arguments’ empirical hypotheses really have the claimed philosophical implications?) as well as on empirical ones (Are the hypotheses really supported by the available scientific evidence?). Lessons from these debates do not always straightforwardly generalize to attempted empirical justifications in political philosophy.^2^ Yet, compared to most other areas of philosophy, empirical approaches to political philosophy have so far prompted significantly fewer critical analyses (for three notable exceptions see Allard and Cova, 2023; Hassoun, 2016; Lindauer, 2020).

The aim of this paper is to contribute to alleviate this situation. More specifically, I attempt to facilitate empirical political philosophy by identifying some of the main pitfalls that it can involve, thus making it easier to avoid them in the future. This will be done by way of example. Specifically, I will consider one particular philosophical argument from public opinion, namely Miller’s case for needs-based justice, as it has been explained in detail in *Principles of Social Justice* (1999) and recently been updated in “Needs-based Justice: Theory and Evidence” (2020). Taking Miller’s account of why and how evidence for public opinion matters for granted, I will ask whether his application of this account is compelling, and draw lessons for empirical political philosophy in general.

Miller’s argument makes for a good case study for at least three reasons. First, Miller is one of the most explicit and influential defenders of an empirical approach to political philosophy. At various places he makes clear that, in his view, “empirical evidence should play a significant role in testing a normative theory of justice” (1999: 51), and more specifically, that any such theory is “to be tested, in part, by its correspondence with evidence concerning everyday beliefs about justice” (1999: 51) (see also 2018, 2020) — a methodology that has attracted much attention, including a new peak just recently (Baderin, 2018; Busen, 2018; Schramme, 2018; Ulas, 2018; see also, e.g., Swift, 2003).

Second, Miller’s argument for needs-based justice has been among the most important parts of his work. Not only does it figure prominently in many of his general publications on justice, including the above-mentioned *The Principles of Social Justice*; the argument has also significantly shaped his co-authored Stanford Encyclopedia of Philosophy entry on “Needs in Moral and Political Philosophy” (Brock & Miller, 2019), has recently been picked up again and further defended and refined by him (2020), and has played an important role in some of his arguments in applied political theory, for example, in supporting his views on immigration and human rights.

Third, Miller’s argument for needs-based justice is also one of the most plausible instances of empirical political philosophy. He has defended his methodology against a number of objections (e.g., Miller, 1999, 2018), and has been extremely careful and cautious in his claims about what science actually tells us about public opinion — more careful and cautious than some other empirical philosophers have been. In focusing on Miller’s case for needs-based justice one hence does not risk fighting a straw man. If even an argumentation as advanced as his is subject to some worry then many other instances of the empirical approach to political philosophy will likely be affected by it as well.

My investigation of Miller’s argument and the proper ways of doing empirical political philosophy will consist of three parts. In the first part I will review Miller’s argument, in particular, his methodology, his philosophical claims and the empirical hypotheses that he appeals to in justifying these claims. In the second part I will raise four worries about the relevance, quantity and philosophical implications of the studies that Miller appeals to in support of his empirical hypotheses. On the basis of these considerations, I will finally attempt to formulate some general recommendations for how to do empirical political philosophy.

## Miller’s Empirical Argument

The first step in evaluating Miller’s empirical argument for needs-based justice consists in properly understanding it. In this Section I will therefore first introduce Miller’s methodology, then the philosophical claims that he attempts to establish and finally the empirical evidence that, in his view, support these claims.

### Miller’s Methodology

Miller distinguishes two main reasons why “what the people think” is relevant to theorizing about justice (see in particular 2020; and also 1999, 2008, 2018). For ease of reference, I will call them the “theoretical justification” and the “practical justification”.^3^

#### The Theoretical Justification

In justifying his philosophical claims about justice, Miller employs a version of the method of reflective equilibrium (see in particular 1999), as it was initially proposed by Rawls (1999). That is, he attempts to bring coherence to his beliefs at three levels. The first level are his considered justice judgments about particular cases. The second level are the principles of justice that govern these judgments. And the third level are non-normative and normative background theories, such as about what it means to be a person or what morality generally aims to achieve.

By “considered judgments” Rawls means “those judgments in which our moral capacities are most likely to be displayed without distortion” (1999: 42). More specifically, he suggests that considered justice judgments are judgments that fulfill the following three conditions: (1) they are pre-theoretical in the sense that they have not been inferred from principles, and are thus able to serve as independent evidence^4^, (2) they are reliable in the sense that they have not been (overly) influenced by factors that are irrelevant to their truth, and (3) they are held with some level of confidence. Miller seems to use the term in an almost identical way (e.g., 1999, 2018, 2020).

In Miller’s view, empirical evidence about public opinion is relevant to justifying philosophical claims about justice in that it can uncover what principles of justice people endorse (1999; see Baderin, 2017, 2018). This, in turn, can help the philosopher to assess the extent to which their own judgments about justice qualify as considered in the sense of being pre-theoretical and reliable. For example, if a philosopher learns that his or her justice judgment about a particular case conflicts with a widely held principle this should lead the philosopher to reexamine whether this judgment may have resulted from an adjustment to a principle that he or she holds (failure of the pre-theoretical condition) or from his or her particular social position, such as his or her disproportionately liberal surroundings (failure of the reliability condition).

[…] attending to empirical evidence about how non-philosophers think can help philosophers to guard against deficiencies in their own reasoning processes. […] their social position biases them in favour of certain views and against others […]. This means that they will often regard as self-evidently true, and therefore as touchstones for normative reasoning, moral or political judgments that people in the wider society are likely to reject. Another [reason] is that philosophers are inevitably tempted to adjust what they take to be their “pre-theoretical” judgments to fit the theoretical positions they have already arrived at independently. (2020: 274–275)

#### The Practical Justification

In addition, Miller thinks that considering public opinion is also important for a practical reason (2003, 2008, 2020). The purpose of theorizing about justice, in his view, is to guide us in dealing with practical problems that occur in the (non-ideal) world that we in fact inhabit. For such guidance to be possible philosophers need to make sure that most lay people can be brought to accept their principles of justice. For example, if the majority of citizens took desert to be central to justice, and their minds could hardly be changed about this, then principles which deny desert’s significance would have to be rejected on grounds of being politically infeasible.

According to Miller, evidence about public opinion is also philosophically relevant in that it can help philosophers to assess which principles of justice could become action-guiding, and hence could be valid.

[…] the final aim of normative theorising as carried out by philosophers is to provide practical guidance on matters of moral or political concern. When such a theory is presented, the principles it contains must, therefore, be ones that people might actually come to embrace and act upon. But if so, they must be accessible to the relevant agents, which means that there must at least be a bridge between these principles and the beliefs (about justice and so forth) that people already hold. […] So, it is important to find out, at least as a starting point, what these beliefs actually are. (2020: 274)

In sum, then, Miller argues that in theorizing about justice there is both a theoretical reason for considering empirical evidence about public opinion (testing whether one’s judgments are considered) and a practical reason (testing whether one’s principle can be action-guiding).

### Miller’s Principle

On the basis of the above methodology, Miller has argued for three principles of justice: a principle of equality, a principle of desert and a principle of need (1999). Below I list and briefly explain what appear to be Miller’s main philosophical claims relating to the principle of need, as discussed in his 2020 chapter.

1. *Justice claims to the satisfaction of needs are distinct from justice claims to the satisfaction of preferences.* (see 2020: 280)

This claim says that when it comes to justice, needs-based claims are not reducible to claims to the satisfaction of preferences.

(C2) *Needs-based claims of justice have priority over other justice and non-justice claims but not absolute priority.* (see 2020: 280)

According to this claim, even if not all people’s needs have been fully satisfied, societies can still be permitted or obliged to distribute goods in ways that rather promote equality, desert, efficiency, etc.

(C3) *The weight of needs-based justice claims depends on (a) the nature of the good that is distributed, (b) the context, i.e., the urgency of distribution and the relationship between provider and recipient, and (c) one’s personal responsibility for being in need.* (see 2020: 281–283)

Miller argues that distribution according to need most obviously has priority when it comes to certain kinds of goods; in particular, goods that can *only* be used to satisfy needs, such as wheelchairs and medicine. The moral weight of needs satisfaction also depends on the context. More specifically, we should give more weight to satisfying needs if doing so is urgent and when certain kinds of personal relationships hold between the provider and the recipient of the good. Finally, Miller argues that people’s claims to need satisfaction become weaker if they are themselves responsible for their state of deprivation.

(C4) *Justice requires satisfying needs in relation to people’s (a) absolute level of need, (b) relative level of need, and (c) rate at which they can convert resources into need satisfaction.* (see 2020: 284)

Under ideal circumstances societies would fully enable all members to meet their needs. Unfortunately, in the real world, some societies lack the resources or motivation to fully comply with their needs-based duties. This introduces the need for principles that guide trade-offs. According to Miller, resources for needs satisfaction should be distributed according to people’s absolute level of need (i.e., how far they are below the level of sufficient need satisfaction), people’s relative level of need (i.e., how much lower or higher their degree of need satisfaction is in relation to a comparison class) and the rate at which people can convert resources into need satisfaction (i.e., what amount of resources is required for increasing need satisfaction by a given level).

(C5) *No single needs-principle of justice can account for all three of the factors listed in C4.* (see 2020: 284–290)

Any valid principle of just distribution according to need, Miller argues, must account for all three of the above factors. Several needs-based principles that have recently been proposed appear to fail to do so, in particular principles of minimizing total neediness, of strict priority for the neediest, of weighted priority, of effectiveness, and of overall and comparative need-satisfaction. This leads Miller to conclude that needs-based justice cannot be reduced to a single principle but is instead “deeply pluralistic in nature” (2020: 275).

### Miller’s Evidence

In the preceding Sub-Section I outlined Miller’s main philosophical claims about needs-based justice. He argues that, given his above-described methodology, C1 to C4 are supported by the following corresponding empirical hypotheses:

1. *Lay people believe that justice claims to the satisfaction of needs are distinct from justice claims to the satisfaction of preferences.* (see 2020: 280)
2. *Lay people believe that needs-based claims of justice have priority over other justice and non-justice claims but not absolute priority.* (see 2020: 280)
3. *Lay people believe that the weight of needs-based justice claims depends on (a) the nature of the good that is distributed, (b) the context, i.e., the urgency of distribution and the relationship between provider and recipient, and (c) one’s personal responsibility for being in need.* (see 2020: 281–283)
4. *Lay people believe that justice requires satisfying needs in relation to people’s (a) absolute level of need, (b) relative level of need, and (c) rate at which they can convert resources into need satisfaction.* (see 2020: 284)

In support of H1 to H4 Miller (e.g., 1999, 2020) appeals to a number of scientific studies, mostly from experimental economics and, to some smaller extent, from social psychology. In these studies participants are typically asked to allocate some good (in a just way) among fictitious recipients, some of whom are in need or in greater need than others. From participants’ responses researchers then infer their underlying needs-based (justice) judgments (e.g. Cuadras-Morato et al., 2001; Lamm & Schwinger, 1980, 1983; Yaari & Bar-Hillel, 1984). In addition, a small number of studies have also taken a more direct approach and explicitly asked participants about their judgments about the significance and nature of needs-based justice (e.g., Forsé & Parodi, 2009).

Table 1 lists all of the scientific studies that Miller cites in his discussion of H1 to H4 in his most recent defense on needs-based justice, i.e., his 2020 chapter. I also provide additional information that will be relevant to my investigation of this evidence in the next Section.

**Table 1:** Scientific studies appealed to in Miller (2020) in his discussion of H1 to H4

	Hypothesis	Sample	Category	Needs	Satisfiers	Question
Cuadras-Morato et al. (2001)	H4b	University of Murcia students, Spain; University Pompeu Fabra at Barcelona students, Spain	Basic	Health	Resources for treatment	Prefer
Forsé and Parodi (2009)	H2	Representative samples from 26 EU countries (except Cyprus)	Basic	Food, housing, clothes, education, health	Unspecified	Justice
Gaertner and Schwettmann (2007)	H3c contr	University of Osnabrück, students, Germany; University of Klagenfurt students, Austria; University of Ljubljana students, Slovenia	Basic	Health, food	Helping, financial aid	Prefer
Hassoun (2009)	H4 add	University of Arizona students, US	Basic	Health	Vitamin supplements	Should
Hurley et al. (2017)	H4a, H4b, H4c	Participants from British Columbia, Saskatchewan, Ontario, and Nova Scotia, Canada; McMaster University students, Canada	Basic	Health	Pills	Other
Konow (2001)	H1, H2	Los Angeles area residents, US; Loyola Marymount University students, US	Basic	Food	Bananas (only food on island), money for fighting malnutrition or agricultural development program	Justice
Lamm and Schwinger (1980)	H3b, H3c	High school students [schools unspecified], Germany	Basic (?)	Education	Money for necessary textbooks	Would
Lamm and Schwinger (1983)	H3b qual	High school students [schools unspecified], Germany	Basic (?)	Education	Money for necessary textbooks	Justice
Matania and Yaniv (2007)	H1, H2	Hebrew University of Jerusalem students, Israel	Basic and non-basic (?)	Education	Educational grants	Prefer
Pritzlaff-Scheele and Zaucher (2017)	H4 add	Lab participants at the University of Hamburg (mostly students), Germany	Basic	Minimum consumption	Money (tokens)	Prefer
Schwettmann (2012)	H3c	University of Osnabrück students, Germany	Basic	Housing/health	Fuel for heating	Should
Scott and Bornstein (2009)	H3a qual, H3b	University of California, Davis students, US	Basic	Health, food	Money, prescription medicine, food	Should
Skitka and Tetlock (1992)	H3c	University of California, Berkely students, US; Southern Illinois University at Edwardsville students, US	Basic	Health	AIDS drug	Should
Sondak et al. (1999)	H3b	Students [university unspecified], US	Not needs	Tcket to a lottery for an airline ticket	Points	Prefer
Ubel et al. (2001)	H3c	Prospective jurors in the Philadelphia County Courthouse, US	Basic	Health	Transplantable organs (livers, lungs)	Would
Umgelter et al. (2015)	H4a	Patients [hospital unspecified], Germany	Basic	Health	Liver transplantation	Should
Weiß et al. (2017)	H4 contr	Lab participants at the University of Hamburg (mostly students), Germany	Basic	Housing	Living space	Justice
Yaari and Bar-Hillel (1984)	H1, H4b	Applicants for admission to the Hebrew University, Israel	Basic	Health	Fruits	Justice

Some information about the studies was not reported explicitly in the corresponding papers but had to be inferred by me. The underlying definition of basic needs is explained in Sect. 3.2. In the “Hypothesis” column qualifiers mean that the study has not been appealed to in support of the relevant hypothesis but for other purposes; in particular, “add” means that the study was meant to provide additional information, “qual” means that it was meant to qualify the hypothesis, and “contr” means that it was taken to provide evidence against the hypothesis. In the “Question” column similar wordings (e.g., “choose” and “prefer”; “fairness” and “justice”) were merged. “?” indicates that an interpretation of some aspect of a study is, in my judgment, open to reasonable disagreement. Finally,  in discussing his empirical hypotheses Miller (2020) also refers to two other papers that are not listed in Table 1. Deutsch (1985) is not included because although the book involves a large number of empirical studies, Miller here only refers to the author’s theoretical claims about personal relationships and the needs principle (personal communication). Foa and Foa (1976) is not included as Miller cites it as an overview of resource theory (which is not directly related to and does not build on empirical studies about needs)

Miller is admiringly careful and cautious in his interpretation of the above studies. For example, rather than relying on single studies he considers a number of them; he does not only consider studies that seem to support but also some studies that seem to contradict or qualify his hypotheses; he knows about the surplus value of converging data from different methodologies and researchers; and so on. But still, does the available evidence really suffice to justify H1 to H4? And do H1 to H4 really justify C1 to C4 in the way suggested by Miller if we take his methodology for granted?

## Worries About Miller’s Argument

Most commentators who have so far engaged with Miller’s empirical arguments about (needs-based) justice have primarily addressed his methodology (e.g., Baderin, 2018; Busen, 2018; Schramme, 2018; Swift, 2003; Ulas, 2018). Here, in contrast, I will take this methodology for granted. Not only do I consider it to be reasonably plausible^5^; it is also (roughly) representative of the ways in which several other empirical political philosophers have attempted to justify the relevance of empirical evidence about public opinion (see, e.g., Allard and Cova, 2023; Brock, 2005, 2009; Hassoun, 2016; Lindauer, 2020; Wolff and de-Shalit, 2007)^6^, which means that it is well-suited to ground investigations that result in more general recommendations.

My focus in this Section will rather be on whether Miller’s empirical hypotheses are well-supported, and whether they sufficiently justify his philosophical claims (on the assumption of his methodology). I will suggest negative answers to these questions for reasons that have to do with the studies’ (1) samples, (2) materials, (3) quantity and (4) theoretical integration.

### Study Samples

Miller claims that theories of justice should account for “what the people think”. But who are “the people”?

A first worry in relation to this question derives from the fact that public opinion about needs-based justice, and justice more generally, may vary across societies.^7^ Table 1 shows that the studies cited by Miller involve participants from a number of different countries: the US (7 studies = 38.89%), Germany (7 studies = 38.89%), Israel (2 studies = 11.11%), Spain (2 studies = 11.11%), and so on.^8^ Miller aggregates the data from all of these studies and does not relativize his claims about needs-based justice in the sense of limiting their application to only one or several particular countries. His argumentation thus seems to rest on the assumption that people’s (considered) judgments about needs-based justice do not considerably vary across societies. Is this assumption warranted?

In *Principles of Social Justice* (1999) Miller provides some tentative arguments for believing so, citing studies which suggest that judgments about inequality and fairness are stable across various countries, including then-communist Poland (Lissowski et al., 1991), Australia (Jackson & Hill, 1995), South Korea (Bond & Park, 1991), and the Philippines (Cruz-Doña & Martina, 2000) as well as the US (Frohlich et al., 1987; see also Frohlich & Oppenheimer, 1992). That said, I think that it is actually still an open question whether people really endorse the same judgments about needs-based justice across cultures.

Consider, for example, the large-scale European Values Survey (as analyzed in Forsé & Parodi, 2009) that Miller refers to in his 2020 chapter. While this study shows some similarities in values across different countries, it also shows some important differences, including differences with regard to needs-based justice. For example, whereas in Malta 84% of participants agreed that “[g]uaranteeing that basic needs are met for all” is more important for justice than eliminating big inequalities or recognizing merit, in the Czech Republic only 57.4% of participants subscribed to this order of priority (Forsé & Parodi, 2009: 210).

Some more indirect empirical evidence, not considered by Miller, suggests cross-national variation in needs-related justice judgments as well. For example, according to Haidt et al.’s Moral Foundations Theory (Haidt, 2012; Haidt & Björklund, 2008; Haidt & Joseph, 2007), while many people in the West predominantly ground their moral judgments in the values of care, fairness and liberty, other cultures also put strong emphasis on loyalty, authority, and purity.^9^ Since justice judgments are based on the value of fairness, these findings make it plausible that people in Western and non-Western cultures differ in terms of how much weight they give to needs-based justice versus other values.

Finally, much justice-related cross-cultural research has focused on economic games such as “ultimatum games” (Güth et al., 1982), in which one player makes a suggestion as to how to divide a given sum of money, and another player can then either accept the offer (in which case the money is divided accordingly) or reject it (in which case neither player gets anything). While ultimatum games do not necessarily and exclusively invoke judgments about justice, it is assumed that they do so often and to at least some extent.^10^ A metaanalysis by Oosterbeek et al. (2004) as well as several other studies suggest that offer and rejection rates in these games vary significantly across countries. For example, while in the Netherlands less than 10% of offers are rejected, French and Spanish respondents tend to reject almost a third of the offers that are made to them.^11^

There is also a second problem relating to the samples of Miller’s studies. One reason why he believes we ought to draw on “what the people think” is that doing so helps us in assessing the extent to which our own justice judgments are reliable; in particular, whether they might have been unduly influenced by our particular demographic characteristics such as our political orientation (2020; see Sect. 2.1). To spot such influences it seems unhelpful to only consider the judgments of people who share these characteristics. Guarding against operating from within one’s own “bubble” rather requires considering the judgments of people who are *different*.

The inclusion of people with alternative demographic backgrounds is also suggested by Miller’s second, practical justification, according to which justice judgments of lay people must be accounted for because one’s principle of justice needs to be action-guiding, which means that it needs to be such that a sufficient number of people can accept it. But if we only know whether a particular (potentially rather small) subset of the population makes judgments in accordance with the principle (e.g., those who are more liberal, more educated and less religious) then we may not be able to draw reliable conclusions about whether the principle can become widely accepted.

The problem that I am hinting at arises from the fact that most of the studies that Miller appeals to only (11 studies = 61,11%) or mostly (3 studies = 16,67%) involved student participants. It is likely that students on average neither differ considerably from Miller in terms of their political orientation, religiosity, etc. nor are they representative (in respect to these characteristics) of the population at large. For example, students are disproportionately politically liberal (Bailey & Williams, 2016; Henrich et al., 2010); they are, almost by definition, disproportionately highly educated (see Bryant 2012), and they are less religious than the average person (Stolzenberg et al., 2018).

There is also some empirical research that suggests that students’ judgments about justice might not generalize to the population at large. In economic games such as the ultimatum game mentioned above non-student Americans in both cities and the countryside were found to make significantly different decisions than undergraduate students (e.g., Henrich & Henrich, 2007; Carpenter et al., 2005); and college-educated US citizens tend to ground their moral judgments in somewhat other moral foundations than non-college-educated US citizens (Haidt et al., 1993; Jensen, 1997).^12^

Thus, the suspicion stands that Miller may not account for “what the people think” about needs-based justice but rather mainly for what a particular kind of people think: people who tend to share certain important demographic characteristics with himself and who live in particular (mostly Western) countries.

### Study Materials

Let us now turn to the materials that participants of the studies referenced by Miller were presented with. I will argue that these materials were generally such that results about them are not sufficient to support his general hypotheses about needs-based justice, i.e., H1-H4.

The first reason for thinking so concerns the kind of needs that participants were asked about. According to Miller’s definition, for a person to need X “it is necessary for that person to have X if he or she is not to be harmed” (1999: 206–207). This harm does not need to be physical or biological. It can also occur in virtue of the person being prevented from having a minimally decent life in the particular society in which she lives. Thus, Miller acknowledges that in a society such as the one that he is part of, people need a secure home, the opportunity to marry and have a family and even certain kinds of shirts and shoes (1999, 2007), with this set of needs further expanding as one’s standard of living increases.

Table 1 shows the kinds of needs that the studies referenced by Miller addressed. These needs were almost always “basic” in the sense of only comprising physical or biological needs as well as a small subset of societal needs that are required for a decent human life in *any* society.^13^ For example, as quoted above, the European Value Survey required rating the importance of the following needs-related principle of justice: “Guaranteeing that basic needs are met for all, in terms of food, housing, clothes, education, health” (Forsé & Parodi, 2009: 210).

This is worth pointing out because it is not obvious that people’s justice judgments about *basic* needs straightforwardly generalize to non-basic needs as well, and hence to needs in general. For example, people might ascribe significantly less weight to meeting needs such as having the opportunity to marry or having certain kinds of shirts and shoes than to needs for health or food (H2). The weight of such non-basic needs claims might also be thought to depend on different factors (H3), and different criteria for the distribution of resources to satisfy these needs might be endorsed (H4).

In fact, the hypothesis that people differentiate between basic and non-basic needs in their justice judgments receives some plausibility from a study by Matania and Yaniv (2007) that Miller cites.^14^ Matania and Yaniv found that their participants gave more weight to equality as opposed to efficiency considerations the more “basic” the need at issue was. In particular, participants tended to distribute educational grants for English as a second language (more basic educational need) equally among all students. With grants for theater studies (less basic educational need), they prioritized students at the highest level, who were said to improve most.

If one considers the particular needs that the studies referenced by Miller involved, as well as the particular resources that participants could distribute to satisfy these needs, a similar worry emerges. Different needs might be judged differently in terms of justice. For example, why assume that people think that needs-satisfying educational resources should be distributed according to the very same criteria as needs-satisfying fruits or vegetables?

The studies that Miller cites only provide limited evidence about whether this possibility might hold. This is because they only asked participants about a very narrow set of (mostly basic) needs: first and foremost health (11 studies = 61.11%), food (4 studies = 22.22%) and education (4 studies = 22.22%).^15^ Other (basic) needs, such as clothing, housing or physical security were only rarely addressed or not at all. This means that the studies in Table 1 might fail to provide a full or sufficiently nuanced picture of what public opinion about needs-based justice looks like in this respect as well.

A final and even more important worry is that many of the studies referenced by Miller may not have measured public opinion about *justice* (see Törnblom, 1992).^16^ In most studies, after having been presented with distributive options, participants had to choose between these options. For example, they had to rate who should get certain educational resources or certain foods. Only a minority of studies (5 studies = 27.78%) asked participants what distribution they consider to be most *just*. Much more often, they were asked how the relevant goods “should” be distributed (5 studies = 27.78%), how they “prefer” them to be distributed (5 studies = 27.78%), or how they “would” distribute them (2 studies = 11.11%).

It is possible that despite not being asked about their justice judgments, participants’ responses are still explained by such judgments to a high degree. But this degree could also be low. For example, when participants were asked how they “would” distribute certain goods, they might not have been engaged in normative thinking at all but might rather have made predictions about their behavior; when they were asked what they “prefer”, participants might have explored their own psychology; and when they were asked how they “should” distribute goods, they might have been engaged in normative thinking but not necessarily (only) about morality, and not necessarily about justice in particular.

In fact, Miller himself cites a study (Lamm & Schwinger, 1983) that lends some initial support to these alternative explanations. In this study one group of participants was asked to allocate money dedicated to buying necessary textbooks among two persons with unequal financial resources. The other group, in contrast, was asked to allocate the money *justly* (“You should perform the allocation in such a way that in your opinion it is as just as possible”; 1983: 207). This justice prompt affected participants’ responses. Compared to the control group, those in the justice condition gave more money to the needier recipient if the recipient was described as an acquaintance, and they gave less money to this recipient if the recipient was described as a friend (Lamm & Schwinger, 1983).

### Amount of Evidence

In the previous two Sub-Sections I have argued that Miller’s evidence for his hypotheses about people’s needs-related justice judgments is rather weak. That said, how well-supported empirical hypotheses are does not only depend on the strength of the evidence that each of a number of individual studies provides; it also depends on the amount of these studies. A lot of weak, suggestive evidence can still add up to a reasonably strong case, especially if many of the studies were conducted by different labs/researchers and were based on different experimental designs. So there may still be hope that we are justified in believing in H1 to H4 on the basis of Miller’s evidence after all (despite the problems that have been pointed out).

Table 1 shows that in his 2020 chapter Miller appeals to no fewer than 18 empirical papers in discussing his hypotheses. However, this total number begins to look less impressive if we consider that some of the referenced studies have been conducted by the same labs/researchers (Lamm & Schwinger, 1980, 1983; as well as Gaertner & Schwettmann, 2007 and Schwettmann, 2012) and that five studies were not supposed to support his hypotheses but only to qualify them, to provide supplementary information about them or are, in fact, discussed as counterevidence (Gaertner & Schwettmann, 2007; Hassoun, 2009; Lamm & Schwinger, 1983; Pritzlaff-Scheele and Zaucher 2017; Weiß et al., 2017).

Any assessment of the quantity of Miller’s evidence must also consider the worry raised in Sect. 3.1. Perhaps what people think about needs-based justice varies so much that in determining public opinion’s significance and nature for any particular country we may only draw on studies that were conducted in this very country. In this case even Miller’s evidence for the US and Germany — the countries that were most often featured in the studies — would be sparse (7 studies from each of these countries across all of H1 to H4, with some of them also being subject to the limitations that were pointed out in the previous paragraph).

Looking at Miller’s hypotheses in detail, it emerges that while some of them are quantitatively better supported by the studies listed in Table 1, others are less so. The latter is particularly true for H4, the hypothesis that lay people believe that justice requires satisfying needs in relation to people’s (a) absolute level of need, (b) relative level of need, and (c) rate at which they can convert resources into need satisfaction. This hypothesis plays a central role in Miller’s argumentation. It is not only used to justify C4 but also C5, i.e., his endorsement of pluralism about needs-based justice. Yet, Miller only appeals to one single study in support of H4, namely Hurley et al. (2017).^17^ Moreover, this study’s relevance is subject to worries that even go beyond those discussed in the preceding Sections.

To begin with, Hurley et al. did not ask participants how to distribute goods among needy recipients most justly; they rather asked how needy they think recipients in given scenarios were in the first place (“which person in the scenario has the *greatest need* and which has the *least need*”; 2017: 121, emphasis original). But the question of how to justly distribute according to need and what needs are must be treated as distinct. One can believe, for example, that need is defined by the extent to which a person falls short of a certain baseline of well-being (absolute need) but still think that distribution according to need requires prioritizing those who most effectively convert resources into need satisfaction (conversion rate).

Miller cites Hurley et al. (2017) as supporting that people endorse distributions according to (a) absolute need, (b) relative need, and (c) the rate at which persons can convert resources into need satisfaction. Another problem with the study’s relation to H4 is that it did not test for participants’ judgments about relative need at all. It only prompted judgments about absolute need (roughly equivalent to what is called “baseline health status” in the study) and conversion rate (roughly equivalent to “resources required to exhaust benefit”). The study revealed that absolute need had by far the most consistent and largest influence on whether participants considered something to be a need. Conversion rate — just as ability-to-benefit, another factor that was tested — only had a small effect.^18^

Suppose I am right that in addition to the problems pointed out in the previous Sections, the quantity of Miller’s evidence for H1 to H4 is not fully sufficient as well (especially when it comes to H4). At this point it is time to turn to a reply that may have been in the back of the reader’s mind for quite a while. That Miller *himself* does not cite a sufficient number of studies that are strongly supportive of his hypotheses does not mean that *there are no* such studies. Relevant studies may still have been conducted; Miller just might not have cited them.

This is, of course, a valid point. However, as far as I can see, Miller’s review of the relevant literature has been relatively thorough. The amount of studies that have addressed needs in the context of justice is low. Moreover, most additional studies that might be interpreted as relevant only pertain to a small proportion of his hypotheses; in particular, to H3c, i.e., the hypothesis that a person’s responsibility for being in need reduces their claim to need satisfaction (see, e.g., Cappelen et al., 2013; Diederich & Schreier, 2010; Farwell & Weiner, 1996; Betancourt, 1990), and to H2, i.e., the hypothesis that lay people believe that needs-based claims of justice have priority over other justice and non-justice claims but not absolute priority (e.g., Adriaans et al., 2019; Hülle et al., 2018; as well as studies based on the above-mentioned Frohlich/Oppenheimer/Eavy research paradigm).

Note also that all of these additional studies suffer from some of the relevancy problems that were discussed in previous Sections as well (and from some additional problems). So while H2 and H3c are certainly better supported than Miller’s other hypotheses, and especially better than H4, there is reason to be cautious even about them.

### Theoretical Integration

In the preceding Sections I have raised several worries about Miller’s evidence for his hypotheses about needs-related justice judgments. But suppose that H1 to H4 could be established. The last worry that I would like to consider is that these hypotheses could still not be used to justify philosophical claims about needs-based justice in the way that Miller has done with regard to at least some of them.

As shown in Sect. 2.1, Miller’s stated methodology for how to integrate empirical information about public opinion in theorizing about needs-based justice is rather complex and indirect. In actual practice, however, he sometimes seems to diverge from this methodology; he seems to take the fact that the folk endorses some philosophical claim about needs-based justice to justify this claim without much further argument. For example, in his recent chapter on needs-based justice (2020) he appears to have moved directly from H1 (Lay people believe that justice claims to the satisfaction of needs are distinct from justice claims to the satisfaction of preferences) to C1 (according to which justice claims to the satisfaction of needs are *in fact* distinct from justice claims to the satisfaction of preferences).

[…] philosophers sometimes doubt whether any real distinction can be drawn between people’s needs and their desires. But we find that in popular understandings of justice, needs are indeed distinguished from preferences and desires, and given special weight. Here is one survey experiment designed to test whether people’s distributive choices vary depending on whether they are confronting differences in need or differences in pleasure or satisfaction (Yaari & Bar-Hillel, 1984). (Miller, 2020: 280)

In addition to the empirical studies referenced in the above quotation and in the subsequent paragraphs, no arguments are presented that relate these studies to C1. So C1 might almost exclusively be based on his views about public opinion.

In what follows I will explain in more detail how Miller's practice of relatively direct inference conflicts with both his theoretical and practical justification for the philosophical relevance of what people think about justice.

Miller’s theoretical reason for why researchers should consider people’s principles of justice is that doing so can help them to assess the extent to which their own judgments qualify as considered in the sense of being pre-theoretical and reliable. This means that once all evidence about public opinion is on the table the theorist is meant to examine his or her own judgments in light of it. The result of this procedure may well be a dismissal — rather than a vindication — of (aspects of) public opinion. For example, the theorist may find that even though he or she differs from the folk in judging that needs-based claims of justice do have absolute priority over other justice and non-justice claims, his or her judgment has not been derived from theories that he or she holds, and has not been influenced by factors that are irrelevant to the judgment’s truth. In this case the theorist might be justified to privilege his or her judgment over that of the folk.

As far as I can see, Miller often does not report on this additional argumentative step. One potential explanation for this is that many of his own judgments about needs-based justice are in line with the folk’s principles. This explanation is supported by the fact that in his 2020 chapter there is one minor case in which Miller departs from public opinion, which is explicitly noted (suggesting that he would have explicitly acknowledged other divergences as well).^19^ Miller also defends the folk’s judgments about principles in terms of their reliability. He argues that participants of the studies that he referenced are unlikely to have suffered from egoistic bias because in these studies they had nothing at stake; they were compensated for their participation regardless of their responses and could not gain or lose in any other way from choosing particular options either (see Miller, 1999).

This defense of the reliability of (needs-related) public opinion may be criticized.^20^ But that is not the main issue here. The observation that I would rather like to draw attention to is that even if Miller’s judgments about needs-based justice were to be consistent with the folk’s principles in almost every instance — which would seem rather striking —, and even if these judgments were fully pre-theoretical and reliable, his theoretical justification would still require that he further processes these judgments in other ways too before he can justifiably endorse them. This is due to his commitment to the method of reflective equilibrium (see Sect. 2).

According to the method of reflective equilibrium, beliefs about principles of justice are justified to the extent to which they cohere with considered judgments about particular cases and background theories. The process of maximizing coherence on these levels may require a theorist to diverge from his or her justice judgments or the principles that initially seemed to best explain them — even if they have been successfully checked against public opinion. Again, at least on an explicit level, Miller does not seem to engage in this kind of coherentist back-and-forth reasoning.

Finally, moving directly from what people think to what is philosophically correct is not warranted on the basis of Miller’s practical justification either. This justification requires that people “might actually come to embrace and act upon” a principle of justice (2020: 274). One way of showing that the public might embrace and act upon a principle is of course to show that they *actually* embrace it and act upon it. This is how Miller seems to attempt to justify his claims about needs-based justice at this more practical level. However, in addition to those justice judgments that people actually hold and act upon there may be numerous others that they *might* be brought to hold and act upon, and that, according to Miller’s practical justification, would hence be warranted too.

This reveals another missing step in Miller’s case for needs-based justice. On the basis of his practical justification it does not suffice to just consider empirical evidence about the current content of public opinion; one would also have to consider to what extent and how this opinion may shift (as investigated by research on moral and non-moral motivations, the effectiveness of public education campaigns, etc.). If people turned out to be at least somewhat open to reason or persuasion — and there is reason to believe that they are (e.g. Robinson, 2013) —, this might significantly extend the range of candidate philosophical claims beyond those endorsed by Miller.

For example, suppose that Miller is right that people believe that the weight of needs-based justice claims depends on people’s personal responsibility for being in need (C3c). This renders the principle justified in a practical sense. However, the opposite principle — namely, that the weight of needs-based justice claims does *not* depend on people’s personal responsibility — might be justified in this sense as well, since it could be the case that if we instructed people about our limited powers of self-governance or the implications of C3c (in terms of the prevention of suffering and death) then they would gradually come to embrace and act on this opposite principle. Further empirical evidence would have to be presented to show that this scenario is unlikely; only then C3c could be said to be at a (significant) advantage in practical terms.

Needless to say, the above worries are contingent on my interpretation of Miller’s stated methodology and actual practice; and such interpretations have proven to be controversial (for discussion see, e.g., Baderin, 2017, 2018; Busen, 2018; Schramme, 2018; Ulas, 2018). I am also only considering needs-based justice here. That is, I do not mean to suggest that Miller may have moved equally directly from empirical hypotheses about public opinion to philosophical claims about equality or desert or other matters. Still, if what I have said above is roughly correct then, at least in some cases regarding needs-based justice, Miller has not stayed fully true to his own methodology.

## Lessons for Empirical Political Philosophy

In the previous two Sections I investigated Miller’s renewed empirical case for a needs-based principle of justice. It turned out that Miller’s hypotheses about needs-related public opinion are not well-supported. While there is some weak evidence in favor of H2 and H3c, we know very little about the factors that determine people’s judgments about the particular weight of needs claims (over and above responsibility) and about the particular pattern of distribution of needs-satisfying resources in varying contexts. Moreover, even if true Miller’s hypotheses would not support his philosophical claims about needs-based justice as directly as his actual practice suggests.

This skepticism about Miller’s argument is a philosophically important result in and on itself. After all, his justification of and claims about need-based justice have been highly important (see *Introduction*). In this Section, however, I will suggest that the aforementioned worries are not peculiar to Miller; they similarly apply to other theorists’ arguments in empirical political philosophy as well, especially to arguments pertaining to the concept of justice. It hence seems worthwhile to derive more general recommendations from my above investigations. Such recommendations could be helpful both in assessing existing research and, even more so, in improving future work (given that empirical political philosophy has only just taken off and will likely become increasingly established).

### Check (and Potentially Account) for Inappropriately Narrow (and Broad) Samples

In Sect. 3.1 I argued that the studies that Miller appeals to do not provide evidence about “what the people” think but mostly only about what a *particular kind* of people think, namely students from Western countries. Samples of this kind abound in the empirical research that has been referenced by political philosophers; see, for example, the student-samples of the studies by Frohlich et al. (1987) as well as their various cross-cultural replications (as referenced, e.g., in Brock, 2005, 2009; Cropanzano et al. 2011; Miller, 1999; Mulligan, 2018). Since there is reason to believe that students from Western countries are not representative of philosophers’ target populations (typically: a country’s or the world population at large), and as students tend to share many of philosophers’ own demographic characteristics, this limits the evidentiary weight of the respective studies.

One aspect of this problem has recently already started to right itself. Many relevant current empirical studies (e.g., Bruner & Lindauer, 2020; Goya-Tocchetto et al., 2016; Nadelhoffer et al., 2013) no longer draw on samples of students but rather from online crowdsourcing services such as Amazon Mechanical Turk or Prolific Academic, which are more diverse and representative in terms of age, education, wealth and other characteristics (Buhrmester et al., 2011). But this still leaves geographical disparities intact. If empirical political philosophers put forward hypotheses about the normative judgments of people in a general or unqualified sense — rather than only people in the US or in Western cultures — caution has to be exercised with regard to much recent research as well (as is suggested by my considerations in Sect. 3.1).^21^

Conversely, the samples of referenced studies can also be too broad. Some of political philosophers’ empirical hypotheses have only been meant to apply to specific populations, such as in investigations of what explains polarization within particular political systems or disagreements between particular groups (e.g., Viciana et al. 2019). In these cases researchers must take equal care to not rely on research that focuses on other populations.

### Verify Studies’ Relevance for your Empirical Hypothesis (and Consider Conducting Studies yourself)

Another lesson that can be drawn from my investigation of Miller’s argument is the importance of carefully attending to what the referenced studies really measure. The most obvious potential problem concerns the target normative judgments themselves. It is possible that studies that appear to measure these judgments, or that have been reported to measure them by others, actually do not do so. For example, they may not ask participants what is *just* (or fair, or democratic, etc.) but only what they “prefer” or “would choose” (Sect. 3.2)^22^, in which case inferences about judgments about justice (or these other normative concepts) are sometimes not (fully) warranted without further argument.

Several other aspects of studies’ experimental design can decrease their relevance for political philosophers’ empirical hypotheses as well, i.e., the amount of justification that they confer on these hypotheses. For example, the studies might measure wrong aspects of the target judgments (see distribution according to need vs. the nature of needs in the context of Hurley et al., 2017, Sect. 3.3) or they may operationalize particular constructs in an imperfect or incomplete way (see studies’ potential contingency on the particular needs that they asked participants about, Sect. 3.2). I take it to be likely that in the end much research that at first glance seems relevant to a given empirical hypothesis of philosophical interest in fact is not or only in a limited sense.

As an example, take Brock’s (2005, 2009) interpretation of Frohlich et al. (1987). In this study groups of participants were asked to choose among income distributions after they were placed behind a “veil of ignorance”, in the sense of being shielded from knowledge of their income class. It turned out that under these circumstances 78% of groups preferred to maximize the average income with a floor constraint that guaranteed a certain minimal income. Brock (2005, 2009) suggests that this result supports that people widely endorse a principle of justice according to which all people should be able to meet their basic needs. However, endorsing some minimal amount of income is in fact not equivalent to endorsing basic needs satisfaction, as, for example, some persons require highly expensive medical care to be able to meet their basic biological needs — which even a rather high income may not guarantee.

Problems such as these highlight the significance of philosophically informed studies along the lines of experimental philosophy. Experimental philosophers conduct or contribute to empirical studies that have been designed for the express purpose of advancing some philosophical debate (see Knobe & Nichols, 2017). This leads to data that is generally much more philosophically relevant than data that has been gathered for independent scientific means. In recent years more and more experimental philosophy studies have been conducted on concepts such as knowledge, intentional action or causation. Curiously, political philosophy has so far been largely exempted from this trend. As Hassoun (2016: 234) points out in a review article: “[t]here is very little experimental political philosophy proper” (for a similar assessment see Allard and Cova, 2023; for some exceptions see, e.g., Aguiar et al., 2013; Bauer et al. 2022; Bruner & Lindauer, 2020; Freiman & Nichols, 2011; Hassoun, 2009; Inoue et al. 2022; Pölzler et al. 2024).

Assuming an empirical approach to political philosophy, experimental philosophy could significantly advance many debates in this area. In the case of Miller, for example, studies that involve less liberal, less educated, more religious, etc. participants and that ask questions about the justness of distributions of resources for the satisfaction of a variety of different basic and non-basic needs would allow for a more thorough assessment of his claims about needs-based justice. There could accordingly be much value in political philosophers increasingly taking the leap from “empirical” to “experimental”, and starting to contribute to scientific research themselves.

### Adjust your Confidence to the Amount of Available (Valid, Reliable, Independent and Converging) Empirical Evidence

My discussion of Miller’s argument (Sect. 3.3) and the above recommendations have repeatedly concerned whether there exists enough evidence in support of particular empirical hypotheses. How much is “enough”? I do not think there is a ready-made simple substitute to well-developed judgment here. The answer will depend on the referenced studies’ level of validity (the extent to which they measure what they were supposed to measure) and reliability (the extent to which their results are reproducible); on the extent to which they have been conducted by independent researchers and labs; on the extent to which results from different methods converge; on the amount of conflicting evidence; and so on. That said, single studies or small numbers of studies typically do not allow for confident conclusions. This is because no single study will ever be fully comprehensive and fully valid.

As an example, take once again the study by Frohlich et al. (1987; see also Frohlich & Oppenheimer, 1992) which has been referenced by political philosophers particularly often, and sometimes as the only or one of very few references for certain philosophical claims (e.g., Brock, 2005, 2009; Mulligan, 2018). I have already explained that under this study’s somewhat idealized circumstances 78% of groups preferred to maximize the average income with a floor constraint that guaranteed a certain minimal income. According to Frohlich et al. themselves, as well as to a number of political philosophers (e.g., Cropanzano et al., 2011; Miller, 1999; Mulligan, 2018), this first and foremost amounts to a refutation of Rawls’ difference principle, according to which inequalities are only permissible if they favor society’s least well-off.

However, even though Frohlich et al.’s study is admiringly well-done, its validity (in relation to its stated aims) can be doubted (see, e.g., Hirose, 2014). For example, the study only investigates people’s judgments about the justness of distributing *income*, while Rawls claimed that his difference principle applies to all „primary goods“, including wealth, powers, liberties, and the social bases of self-respect. It is possible and plausible that if participants had been asked how to justly distribute this whole set of goods behind a veil of ignorance then they might have endorsed other principles than they did in Frohlich et al.’s income-only setup (and perhaps even the difference principle).

In response to worries such as these it might be pointed out that Frohlich et al.’s study has been repeatedly replicated; including, as mentioned above, in several different countries (e.g., Jackson & Hill, 1995; Lissowski et al., 1991). These replications have also been referenced by some political philosophers (e.g., Cropanzano et al. 2011; Mulligan, 2018). However, it is important to note that replications only testify to the original study’s *reliability*; they do not show that the original study (or any of the replications) were valid in the sense of having measured judgments about the difference principle in a Rawlsian sense. In other words, even if an effect is found on and on this does not by itself do anything to vindicate the researchers’ interpretation of this effect.

Thus, those political philosophers who have argued that empirical findings put pressure on the difference principle need to come up with additional evidence in favor of their claim, beyond that gathered by Frohlich et al. (1987) (among other things, evidence that does not only reflect judgments about the distribution of income but also about other primary goods). The important general take-away is that for an empirical hypothesis to be properly supported it needs to be in line not only with a small but a substantial number of studies, and studies that are highly valid and reliable. The theorist must identify a well-established general trend in the literature and show how this trend supports their hypothesis.

### Philosophically Integrate Empirical Hypotheses according to your Actual Methodology

My final worry about Miller’s argument concerned the integration of his empirical hypotheses into his philosophical theorizing. This again looks like a common worry about empirical political philosophy. If asked no philosopher would subscribe to the view that we should just adopt whatever the folk thinks about normative concepts such as justice, liberty, power, etc., unless perhaps these judgments were made in highly idealized circumstances. Yet, some empirically informed arguments give the impression that something like that may actually be happening. Political philosophers sometimes seem to be very quick to take the fact that public opinion sides with their philosophical claims as evidence for these claims.

Besides Miller, this problem may, for example, be exemplified by Hassoun (2009). Hassoun claims that principles of distribution can be justified by showing that “well informed and appropriately impartial people (perhaps placed in something like an original position) would accept them” (2009: 261). Then she argues that her favored needs-related principle is superior to an alternative principle by Miller because it is more consistent with the results of an empirical study that she conducted. However, judging from the description of this study, it is not clear that the participants were indeed placed in suitably ideal circumstances. So again, the move from “people think that x is just” to “x is just” might have been more direct than it should have been by the researcher’s own lights.

Conversely, political philosophers can also fail to stay true to their stated methodologies by giving *less* weight to evidence about public opinion than they promised. This problem may show in Wolff and de-Shalit’s (2007) attempt to come up with a list of central capabilities that can be used to measure disadvantage. On the basis of epistemic, feasibility- and democracy-related reasons Wolff and de-Shalit argue for a method of public reflective equilibrium, in which both the philosopher’s and the folk’s judgments about particular cases and about principles need to be brought into coherence (for a discussion of similarities and differences with Miller’s theoretical justification, as explained in Sect. 2.1, see Baderin, 2017). Then they put this method into practice on the basis of capabilities-focused interviews that they conducted in the UK and Israel.

This is, in my view, a promising approach of integrating empirical evidence into theorizing about political philosophy. That said, it seems to me that in employing their method of public reflection equilibrium Wolff and de-Shalit considered people’s opinions to a lesser extent than they had in fact committed themselves to. Even in cases in which their interviewees disagreed pretty strongly that some particular capability should be regarded as morally weighty Wolff and de-Shalit (2007) still held on to these capabilities as valid measures of disadvantage. For example, this is how Wolff and de-Shalit report participants reactions to “Practical Reason. Being able to engage in critical reflection about the planning of one’s life”:

Here, more than elsewhere, the suspicion of the philosophers’ intellectualist bias raised its head. Some interviewees were bemused about how this could be considered an important functioning when so few people appear able to achieve it. Another pointed out that what seems to be at stake here is an idea of self-determination, yet that need not take the form of second order critical reflection. (Wolff and de-Shalit 2007: 53–54)

Nevertheless, the “Practical Reason. Being able to engage in critical reflection about the planning of one’s life”-item made it onto Wolf and de-Shalit's final list of capabilities.

Another capability that was met with resistance on the part of Wolff and de-Shalit’s participants, yet finally vindicated, was “Other species. Being able to live with concern for and in relation to animals, plants, and the world of nature.”

Most of our interviewees thought this was not a very important functioning. One, who works with disabled persons, laughed in embarrassment and justified her position that this was a luxury, and at any rate, not an important functioning. A disabled interviewee said this was not a condition for a good life. On the other hand, another interviewee suggested that relationships with other species were extremely important, although not many people acknowledge this, adding that as long as they did not acknowledge this there was nothing to do about it, so the state was not obliged to see that people achieve it. (Wolff and de-Shalit 2007: 57)

Since people disagreed so considerably with these two capabilities being central, it seems to me that a proper application of Wolff and de-Shalit’s method of public reflective equilibrium would have required to either dismiss the capabilities as measures of disadvantage or to develop strong coherentist arguments for their inclusion – neither of which was provided.

In this paper I have not argued that any particular way of informing political philosophy by empirical evidence about public opinion is better than others (even though I have expressed some qualified sympathies towards Miller’s methodology). My point here thus only is the following: whatever empirical political philosophers’ preferred methodology looks like, they should take care to actually follow through with it, and to not give considerably more or less weight to what the people think than this methodology requires, or to do so in different ways than it requires.

## Conclusion

In recent years an increasing number of political philosophers have begun to ground their arguments in empirical evidence gathered by science. In this paper I have investigated this novel approach by way of example. The object of my case study was David Miller’s renewed empirical argument for a needs-based principle of justice. I raised four worries about this argument. These worries gave rise to the following corresponding general recommendations for how to do empirical political philosophy:

1. Check (and potentially account) for inappropriately narrow (and broad) samples.
2. Verify studies’ relevance for your empirical hypothesis (and consider conducting studies yourself).
3. Adjust your confidence to the amount of available (valid, reliable, independent and converging) empirical evidence.
4. Philosophically integrate empirical hypotheses according to your actual methodology.

These recommendations are of course incomprehensive, reflecting only some of the pitfalls that lurk in appealing to scientific studies. They are also, in some sense, trivial. I am sure that in the abstract every empirical political philosopher would happily agree with them. Still, the recommendations are not always adhered to in practice, not even by some of the most careful and cautious proponents of the approach. This means that hopefully they will still prove to be at least somewhat helpful in further advancing the promising endeavor that is empirical political philosophy.

## Footnotes

1 Most commonly, contemporary theorists have used the method of “reflective equilibrium” which draws, among other things, on the theorist’s own considered judgments or intuitions about justice (see Sect. 2.1).

2 This is the case, for example, because these justifications are often based on the method of reflective equilibrium, which is to some extent peculiar to normative theory, and because political philosophers have not only appealed to evidence from psychology but also from experimental economics and other fields.

3 Miller does not use these very labels but explicitly distinguishes between the above two kinds of justification, and often describes the first in terms of epistemic justification (a theoretical endeavor) and the second in terms of solving practical problems (e.g., 2020). The labels are also in line with how other authors have referred to identical or similar distinctions (e.g., Lindauer, 2020).

4 According to Miller (2018), deriving one’s considered judgments from one’s principles or theories would be equivalent to manipulating data for the purpose of justifying an antecedently held hypothesis.

5 The only aspect that I am not sure about is whether Miller is right to focus on lay people’s favored *principles* of justice (rather than their judgments about particular cases). For discussion of this question see Baderin, 2017.

6 While Lindauer focuses on what I have here called the practical justification (see Sect. 2.1), many other authors have attempted to justify the philosophical relevance of public opinion by drawing on the public’s considered judgments about either principles of justice or particular cases relating to justice, which are sometimes treated as input to the method of reflective equilibrium (see the other of the above references; and for an attempt to develop a “public reflective equilibrium” in particular my discussion of Wolff and de-Shalit in Sect. 4.4).

7 If people’s justice judgments varied across society this would, in my interpretation, commit Miller to at least some weak form of moral relativism. Justice would demand something else in one society than in another. For discussions about potential relativistic ramifications of Miller’s theory of justice see Busen, 2018; Swift, 2003.

8 The above percentages do not add up to 100% as some studies involved participants from more than one country.

9 For some recent criticisms of Moral Foundations Theory see Kivikangas et al., 2021; Zakharin & Bates, 2021.

10 That respondents judge certain offers to be unfair is often thought to be the best explanation of why they reject these offers. After all, if they just aimed at maximizing utility (as assumed by the homo oeconomicus model), respondents should accept any offer, regardless of how low.

11 There is also solid evidence that members of small-scale societies in Africa, Amazonia, Oceania, Siberia, and New Guinea behave differently than those of industrialized societies in economic games (Henrich et al., 2005, 2006, 2010). I do not report these studies above as Miller’s theory of justice is only supposed to apply to modern liberal societies.

12 This raises the possibility that even within one particular society there may be no sufficient consensus on (needs-based) justice. For a discussion of this possibility and its implications for Miller’s account see Ulas (2018) and Miller (2018).

13 The above definition of basic needs follows Miller himself, who defines these needs as “conditions for a decent human life in any society” (2007: 181). Note that one study in Table 1, conducted by Sondak et al., did not measure needs at all, given the above definition of needs. In this study allocation decisions by participants determined who would have the chance to win an airline ticket to any destination within the continental US. Need was operationalized as the amount of points lacking to be able to participate in that lottery.

14 The support is likely only weak. This is, among other things, because in the study by Matania and Yaniv participants were not asked about the justness of distributions but only about which distributions they “prefer”, and because it is open to debate to what extent learning English as a second language could be regarded as a (more) basic need. For a more detailed discussion of these worries see below.

15 Note that some studies covered more than one of the above needs.

16 Miller mentions this in *Principles of Social Justice* (1999) but only in the context of the conceptual distinction between justice and non-justice judgments. He does not directly consider the above objection.

17 Miller might reply that in fact three studies that he cited in support of other hypotheses can also be interpreted as supporting aspects of H4, namely Umgelter et al. (2015) with regard to distribution according to absolute need as well as Cuadras-Morato et al. (2001) and Yaari and Bar-Hillel (1984) with regard to distribution according to relative need. But even if these studies are factored in, support of H4 remains fairly low, given the hypothesis’ central importance. Moreover, these additional studies suggest that lay people do not only endorse distributions according to the criteria proposed by Miller but also according to urgency (Umgelter et al., 2015) and ability to benefit (Cuadras-Morato et al., 2001; just as Hurley et al. 2017). This raises the question of why these criteria have not been included in H4 as well.

18 Moreover, surprisingly, the direction of the result varied across participants. While some thought that a larger amount of resources required to exhaust benefit was indicative of greater need, others associated greater need with a lower amount (Hurley et al., 2017).

19 Weiß et al. (2017) found that people give special weight to bringing people up to the basic needs threshold, i.e., the more persons have fully fulfilled basic needs the better. Miller dismisses this finding on the basis of his own intuition about what he takes to be an analogous case: “Suppose, by way of analogy, that I owe one person £200 and another person £400 and I have an indivisible lump sum of £200 to allocate. What would be the argument for paying off the smaller debt in full? I could, of course, say “well, at least I’ve paid off one of my debts”, and that might be satisfying to know, but how could it be what justice requires? My intuition is rather that justice requires reducing the larger debt, or at most is neutral between the two options.” (2020: 289, fn. 12).

20 In light of the pervasive effect of self-interest on moral judgments (e.g., Bocian & Wojciszke, 2014; Epley & Caruso, 2004; Messick and Sentis 1979, 1983) it is plausible that it may still have influenced participants’ judgments in more indirect ways. Moreover, justice judgments cannot only be unduly influenced by self-interest but by numerous other irrelevant factors as well, such as emotions (e.g. Seidel & Prinz, 2013; Strohminger et al., 2011; Valdesolo & DeSteno, 2006), the way in which information is framed (e.g. Petrinovich & O’Neill, 1996) or the order in which information is presented (e.g. Lanteri et al., 2008; Wiegmann et al., 2012 and Liao et al., 2012). Miller himself also mentions two further potential disturbing factors, namely mistaken assumptions about empirical facts and faulty inferences. Finally, there is also some initial evidence that needs-related consideration in particular might be affected by biases. In a previous study (Pölzler & Hannikainen, 2022) my collaborator and I found that participants in higher income brackets endorsed narrower conceptions of basic needs than those in lower brackets — presumably, because those in higher brackets are more likely to be providers and those in lower brackets are more likely to be receivers of means to needs satisfaction.

21 Experimental philosophers have recently increasingly conducted cross-cultural investigations (e.g., Machery et al., 2017; Rose 2020). Some have interpreted these investigations as supporting the stability of several kinds of philosophical intuitions across different cultures, such as of intuitions about free will, intentional action and reference (Knobe, 2021), while others have contested this interpretation (Stich & Machery, 2022). In any case, it is not clear what these findings imply for intuitions about (needs-based) justice in particular; and these intuitions themselves have not yet been the subject of any cross-cultural experimental philosophy studies.

22 An analogous problem has afflicted much of empirical and experimental ethics (see Pölzler, 2018; Schaich Borg et al., 2006). For example, in many psychological studies on Trolley dilemmas participants were not ask which action is “morally required” or “morally permissible” but rather only which one is “appropriate.”
